# Hardware-Efficient Real-Valued Neural Predistorter for Multimode Power Amplifiers

**DOI:** 10.3390/s26113503

**Published:** 2026-06-02

**Authors:** Luiza Beana Chipansky Freire, Luis Schuartz, Eduardo Gonçalves de Lima

**Affiliations:** Electrical Engineering Department, Federal University of Paraná, Curitiba 81531-980, PR, Brazil; luisschuartz@ufpr.br (L.S.); eduardo.lima@ufpr.br (E.G.d.L.)

**Keywords:** digital predistortion, power amplifiers, multimode power amplifiers, neural networks, real-valued neural network, behavioral modeling, low-complexity design, hardware-efficient DPD

## Abstract

Digital predistortion (DPD) is essential for mitigating nonlinear distortion in radio-frequency (RF) power amplifiers (PAs), particularly in modern multimode transmitters. Among the existing approaches, the neural-network-based DPD reference model adopted in this work is attractive due to its high modeling accuracy and effective predistortion capability. However, its practical implementation is hindered by the computational complexity of the preprocessing stage, which relies on magnitude extraction, phase normalization, and trigonometric operations. Motivated by this limitation, this work proposes a simplified hardware-efficient formulation, derived from an existing real-valued three-layer perceptron (TLP)-based DPD model, for multimode PA linearization. The proposed approach preserves the main characteristics of the reference model while replacing conventional magnitude and phase normalization with a simplified feature representation derived from complex-valued signal products, eliminating square-root, reciprocal, and trigonometric operations. Two configurations are investigated: a single-network formulation and an iterative cascaded structure composed of compact networks trained sequentially. Simulation results demonstrate accuracy comparable to the reference model while reducing computational complexity by up to 34% in multiplications, 25% in additions, and 73.9% in LUT usage, making the proposed approach suitable for FPGA and ASIC implementations.

## 1. Introduction

DPD is a well-established technique for compensating the nonlinear behavior of RF PAs, enabling high-efficiency operation while meeting stringent linearity requirements. However, the increasing complexity of modern wireless systems, particularly those employing multimode and multiband PAs, introduces significant challenges for real-time DPD implementation across different hardware and software platforms.

Neural-network-based behavioral models have demonstrated strong modeling capability for nonlinear systems with memory effects. Nevertheless, their direct application in practical transmitters is often constrained by computational complexity, memory requirements, and real-time processing limitations. In particular, complex-valued signal representations and nonlinear mathematical operations, such as square roots, divisions, and trigonometric functions, can significantly increase processing load and reduce implementation efficiency, regardless of the underlying platform.

### 1.1. Motivation for DPD Simplification in 5G and 6G

Fifth- and sixth-generation wireless communication systems impose unprecedented challenges on the implementation of digital predistortion techniques due to the combined effects of ultra-wide bandwidths, multimode operation, stringent transmission quality requirements, and practical implementation constraints. In 5G New Radio (NR) systems and in envisioned 6G scenarios, signal bandwidths may reach several hundreds of megahertz or even exceed 1 GHz, requiring the DPD to process a spectral region several times wider than the useful signal bandwidth in order to compensate for high-order intermodulation products. For instance, [1] proposed a multi-rate hybrid predistortion scheme that reduces sampling rate and resolution requirements while maintaining linearization performance, demonstrating feasibility for next-generation 6G systems with reduced computational overhead. This spectral expansion demands extremely high sampling rates, which substantially increase computational complexity and energy consumption in digital signal processing platforms [2,3].

The adoption of multimode PAs has become an important strategy for improving efficiency in modern wireless transmitters, particularly under signals with large power fluctuations. In practice, high-PAPR waveforms force the PA to operate most of the time with output back-off relative to saturation, which significantly degrades average efficiency if a single operating condition is used. Multimode operation mitigates this limitation by allowing the amplifier to switch among different operating regimes according to the demanded output power, thereby providing a more favorable efficiency-versus-output-power trade-off over a wider dynamic range. However, when this strategy is combined with wideband or multiband transmission, the linearization task becomes considerably more challenging. Larger signal bandwidths intensify memory effects and frequency-dependent behavior, while high-PAPR excitation increases the probability of transitions among operating modes. As a result, the PA exhibits strongly nonlinear, dynamic, and mode-dependent characteristics, making behavioral modeling and DPD significantly more complex. Under these conditions, conventional DPD approaches often require higher nonlinear orders, increased memory depth, and a larger number of model parameters, further intensifying computational and implementation demands [4].

These challenges are particularly critical in mobile devices, small cells, and distributed radio unit architectures, where constraints on power consumption, thermal dissipation, latency, and cost are dominant design factors. Prior studies indicate that increases in sampling rate and computational parallelism directly impact energy consumption and system efficiency, competing with other essential signal processing tasks such as beamforming, fronthaul processing, and real-time control [5,6]. Even in highly optimized hardware implementations, growing DPD complexity leads to increased silicon area, higher development cost, and more demanding verification and calibration procedures [3].

In parallel, 5G and 6G systems are governed by strict transmission quality requirements as defined by standards such as the 3GPP TS 38.104. These requirements must be satisfied across all operating modes and frequency ranges, leaving limited margin for excessively complex or difficult-to-adapt DPD solutions [7]. Furthermore, advanced DPD architectures often rely on high-rate, low-latency feedback paths, which may be constrained or unavailable in mobile and distributed deployments.

Consequently, the simplification of DPD systems emerges not merely as an optimization strategy, but as a fundamental requirement for practical deployment in modern wireless transmitters. Reducing algorithmic complexity, the number of arithmetic operations per sample, and dependence on high-bandwidth feedback enables lower energy consumption, improved thermal behavior, reduced implementation cost, and enhanced robustness to variations in temperature, load, and operating mode. Simplified DPD models therefore represent a crucial trade-off between linearization performance and practical implementation constraints, making them particularly suitable for next-generation 5G and 6G communication systems.

The objective of this work is to reduce computational and implementation complexity while maintaining effective linearization performance. To this end, a simplified real-valued neural network architecture is proposed for multimode PA linearization, aiming at a cost-effective and scalable DPD solution that can be efficiently deployed across a wide range of digital processing platforms.

### 1.2. Multimode PAs: Characteristics and State of the Art

Multimode PAs have emerged as an effective solution to improve energy efficiency in modern wireless communication systems, including 5G and beyond. Unlike conventional single-mode PAs, multimode architectures are designed to operate in multiple gain regimes, such as low-, medium-, and high-gain modes, which can be dynamically selected according to the instantaneous input signal power level [8]. This reconfigurability enables the PA to maintain high average efficiency under high peak-to-average power ratio (PAPR) waveforms. However, it also introduces significant challenges for behavioral modeling and DPD.

A fundamental characteristic of multimode PAs is the presence of abrupt discontinuities in the amplitude-to-amplitude (AM–AM) and amplitude-to-phase (AM–PM) transfer characteristics [9]. These discontinuities occur during transitions between operating modes, where internal circuit branches are activated or deactivated to modify the gain profile. Conventional behavioral models based on memory polynomials (MP) or Volterra-series expansions generally struggle to accurately represent such sharp nonlinear transitions without a substantial increase in model order, which results in excessive computational complexity and limited suitability for hardware implementation [10].

In addition to static nonlinearities, multimode PAs often exhibit pronounced memory effects. These effects arise from thermal phenomena, bias network dynamics, and frequency-dependent behavior, which become more severe due to rapid changes in the circuit operating conditions across different modes [11]. Consequently, an effective multimode PA model must simultaneously account for nonlinear distortion, memory effects, and mode-dependent discontinuities.

To overcome the limitations of polynomial-based approaches, artificial neural networks (ANNs), particularly TLPs, have been extensively investigated as a state-of-the-art solution for modeling and linearizing multimode PAs. Due to their universal approximation capability, ANNs can accurately represent highly nonlinear and even discontinuous input–output relationships without requiring explicit model segmentation [12].

Recent research has shown that the effectiveness of ANN-based DPD for multimode PAs can be further enhanced through several key strategies. The use of activation functions that jointly process amplitude and phase information has been demonstrated to improve modeling accuracy, especially in the presence of strong AM–PM distortion, leading to lower normalized mean square error (NMSE) values compared to amplitude-only representations [9]. Furthermore, phase-normalized neural network structures have been proposed to simplify the network input representation by removing carrier frequency information, which significantly reduces the computational burden associated with wideband signal processing [13].

From a hardware implementation perspective, recent efforts have focused on reducing the complexity of neural DPD architectures through sparse network representations, parameter selection, and pruning techniques. The trend toward hardware acceleration of neural networks for DPD has intensified recently. Khan et al. [14] provide a comprehensive review of next-generation DPD implementations using hardware acceleration strategies on CPUs, GPUs, FPGAs, and ASICs, highlighting the critical role of architectural simplification in enabling practical deployment of neural-network-based DPD in resource-constrained environments.

These approaches aim to minimize the number of required multipliers, adders, and lookup-table-table-based nonlinear (LUTs), enabling real-time implementation in cost- and power-constrained FPGA platforms with only marginal degradation in linearization performance [9,15]. Overall, the current state of the art emphasizes the development of low-complexity neural-network-based DPD solutions that preserve modeling robustness while addressing the nonlinear, memory-dependent, and discontinuous behavior inherent to multimode PAs. It is important to clearly state that The reported LUT-related complexity refers to lookup-table-based nonlinear function blocks required to implement nonlinear operations and does not correspond to the physical LUT utilization of a specific FPGA architecture or synthesis flow.

### 1.3. Contributions of This Work

This work proposes a simplified real-valued neural network formulation for multimode PA inverse behavioral modeling, with emphasis on reducing implementation complexity while preserving modeling accuracy. The proposed approach is motivated by the increasing computational demands of modern DPD systems operating in multimode and multiband scenarios, particularly when real-time implementation on FPGA, DSP-based accelerators, or other dedicated hardware platforms is considered. It is worth mentioning that the proposed strategy is hardware-agnostic, since it targets the arithmetic formulation of the DPD processing chain rather than optimizations tied to a specific platform. Furthermore, reducing complexity requires not only simplifying the arithmetic operations involved in signal processing, but also avoiding excessively large neural network structures that increase the number of trainable parameters, memory accesses, and multiply–accumulate operations.

The reference real-valued neural DPD formulation considered in this work adopts a relevant signal-domain processing strategy by transforming the input signal and suppressing carrier-frequency-related information, thereby enabling compact real-valued TLP networks to model multimode PA inverse behavior with memory. However, this approach may shift part of the computational burden from the neural network to the feature-extraction stage, which can involve magnitude computation, normalization, reciprocal/division, square-root, and phase-related nonlinear operations. The proposed formulation focuses on simplifying this feature-extraction bottleneck while preserving the modeling rationale of the original representation.

In addition, the proposed approach is designed to maintain a compact neural structure by using simplified input features and low-dimensional TLP models instead of relying on large neural networks to compensate for inefficient signal representations. Therefore, the reduction in implementation complexity is achieved by jointly addressing the signal-processing front end and the neural-model size, providing a formulation that is more suitable for practical real-time DPD implementation.

The main contributions of this work can be summarized as follows:

1. A simplified signal representation for real-valued neural network PA modeling is proposed. The formulation replaces magnitude extraction and normalized phase-difference computations with squared-modulus and direct complex-product features, thereby eliminating square-root, reciprocal, and trigonometric operations and reducing the dependence on LUT/BRAM resources and specialized hardware blocks.
2. A hardware-oriented complexity analysis of the input feature generation stage is presented. The analysis quantifies the number of arithmetic operations required by both the literature model and the proposed formulation, highlighting the reduction in nonlinear operations and the resulting simplification of the hardware pipeline.
3. An iterative cascaded training strategy for multimode PA modeling is introduced. In this approach, multiple TLP networks are connected in cascade and trained sequentially by progressively removing the contributions of previously trained networks from the desired signal, enabling improved signal separation among modes.
4. A complexity analysis of the neural network architecture is provided. The analysis derives closed-form expressions for the arithmetic operations required by the TLP structure and extends them to the cascaded formulation.
5. A complexity comparison between the literature model and the proposed approaches is presented, considering both signal-processing operations and neural network structure. The results demonstrate that the proposed model significantly reduces implementation complexity while maintaining competitive modeling performance.

Overall, the proposed methodology provides a hardware-efficient framework for multimode PA behavioral modeling, enabling accurate real-time implementation with reduced arithmetic complexity and flexible neural network configurations.

The remainder of this paper is organized as follows. Section 2 presents the real-valued TLP-based neural network framework and the literature reference model adopted for comparison. Section 3 introduces the proposed simplified multimode PA inverse behavioral model, including the simplified signal representation, hardware complexity analysis, and iterative cascaded training strategy. Section 4 presents the linearization and complexity results obtained for the evaluated models. Finally, Section 5 discusses the main findings and conclusions of this work.

## 2. Real-Valued TLP-Based PA Behavioral Modeling

This section presents the neural network modeling framework adopted in this work for PA behavioral modeling. The approach is based on a real-valued TLP, which provides a good tradeoff between modeling capability and implementation simplicity. Since the objective of this work is to analyze implementation feasibility not only accurate PA modeling, both the neural architecture and its computational implications must be considered.

First, the structure and mathematical formulation of the TLP are described. Then, the computational complexity associated with the neural architecture is analyzed in terms of arithmetic operations. Finally, the reference model from the literature is introduced, including the signal decomposition strategy and the training procedure adopted in previous studies. This analysis establishes the baseline used for comparison with the simplified multimode PA model proposed in the next section.

### 2.1. TLP Architecture

A multilayer perceptron (MLP) is a feedforward artificial neural network composed of interconnected layers of nonlinear processing units. In this work, is used a TLP. The TLP consists of an input layer, one hidden layer, and an output layer.

Real-valued TLPs are capable of modeling nonlinear systems using real-valued weights and biases. Let $I\left(n\right)={\left[I_{1}\left(n\right),I_{2}\left(n\right),\ldots ,I_{k}\left(n\right)\right]}^{T}$ denote the vector of *k* real-valued input features at sample *n*. The adopted TLP contains *R* hidden neurons and *q* output signals. The weight from input feature *i* to hidden neuron *j* is denoted by $w_{ij}$, and the corresponding hidden-layer bias is denoted by $b_{j}$. The weight from hidden neuron *j* to output *r* is denoted by $v_{jr}$, and the output-layer bias is denoted by $c_{r}$.

With this notation, the hidden-layer pre-activation and activation are given by

(1) $$a_{j}\left(n\right)=\sum_{i=1}^{k}w_{ij}I_{i}\left(n\right)+b_{j},\;h_{j}\left(n\right)=\phi \;\left(a_{j}\left(n\right)\right),\;j=1,2,\ldots ,R.$$

The *r*-th TLP output is then obtained from the linear output layer as

(2) $$S_{r}\left(n\right)=\sum_{j=1}^{R}v_{jr}h_{j}\left(n\right)+c_{r},\;r=1,2,\ldots ,q.$$

Equivalently,

(3) $$S_{r}\left(n\right)=\sum_{j=1}^{R}v_{jr}\;\phi \;\left(\sum_{i=1}^{k}w_{ij}I_{i}\left(n\right)+b_{j}\right)+c_{r}.$$

Thus, the nonlinear activation function is applied only at the hidden layer, whereas the output layer is linear.

The activation function ϕ(·) enables the network to learn nonlinear patterns.

The real-valued TLP-based behavioral model aims to reproduce the nonlinear characteristics of the PA using real-valued weights and biases. The models analyzed in this work explicitly consider PA memory effects, which are characterized by the dependence of the AM–AM and AM–PM responses on past input samples. These effects originate from thermal phenomena and long time constants in the DC bias networks [16].

To incorporate memory effects, past samples of the input signal are presented to the model. The memory depth is denoted by *M* and depends on the dynamic behavior of the PA under analysis.

For real-valued neural network PA models, the complex-valued input signal must be decomposed into real-valued components. Common approaches include decomposition into real and imaginary components or into amplitude and phase components. Previous studies have shown that carrier frequency information should be removed prior to neural network processing to avoid out-of-band distortion and improve modeling robustness [16]. In [17], it is demonstrated that, due to the nature of the PA and the neural network activation functions, carrier frequency information should be removed from the signal prior to network processing in order to avoid out-of-band distortion.

In addition to modeling accuracy, the signal decomposition strategy directly impacts implementation complexity. Therefore, this work proposes a simplified real-valued TLP-based multimode PA model that focuses on reducing the number of arithmetic operations required for real-time implementation.

**Figure 1:**
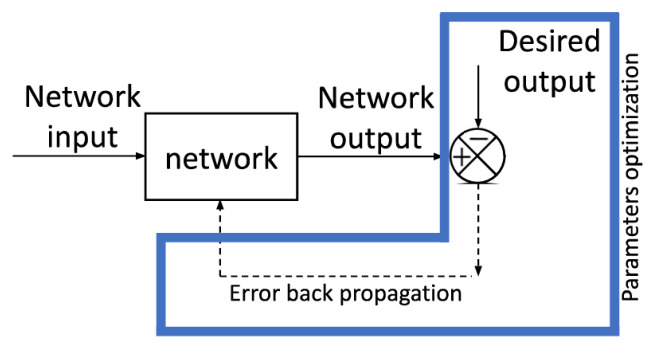
Block diagram that describes the parameter optimization of a neural network mathematical model.

The training process aims to optimize the parameters that describe the system model. Neural networks typically employ the backpropagation algorithm, which iteratively adjusts the network weights and bias values based on the error computed between the network output and the desired signal. In other words, the network learns to reproduce the target signal presented at its output. Throughout this work, references to the network output correspond to the desired signal provided to the network during training. This training procedure is illustrated in Figure 1.

### 2.2. TLP Computational Complexity

In addition to modeling capability, the computational complexity of neural network architectures is a critical aspect when considering real-time implementations of DPD systems. Since PA models operate on high-rate baseband signals, the number of arithmetic operations required by the neural network directly impacts hardware cost, processing latency, and power consumption.

Recent hardware-aware NN-DPD works address implementation complexity from different design levels. Mixed-precision and quantization methods reduce the bit width and memory footprint of the neural model [18]; DeepShift-like approaches replace multiplications in neural layers with bit-shift and sign operations [19]; sparsity and pruning reduce the number of active parameters or operations and can be exploited in FPGA accelerators [20]; and dedicated ASIC/accelerator implementations optimize the physical execution of selected neural architectures [3]. These approaches mainly reduce the cost of neural inference or its hardware mapping, and recent survey-level work has highlighted this as a key direction for next-generation NN-DPD implementation [14]. In contrast, the proposed method acts at the feature-generation level, before the neural network is evaluated. It reduces the cost of generating the nonlinear input features that feed the TLP and is therefore complementary to quantization, pruning, shift-based arithmetic, and accelerator-oriented implementations.

Therefore, it is important to quantify the computational requirements of the adopted TLP structure. This analysis considers the number of arithmetic operations involved in a forward pass of the network, including multiplications, additions, and evaluations of the activation function. These metrics provide a hardware-independent measure of complexity that can be used to compare different neural network configurations.

In this work, all models employ a real-valued TLP composed of an input layer, one hidden layer, and one output layer. The hidden neurons use the hyperbolic tangent sigmoid activation function, whereas the output layer is linear. Let *k* denote the number of real-valued input features, *R* the number of hidden neurons, and *q* the number of output signals generated by the network. Based on the architecture described in the previous subsection, the computational cost of the TLP can be analytically derived as described in the following.

For a single TLP, each hidden neuron computes a weighted sum of the *k* inputs plus a bias term, followed by the nonlinear activation function. Then, each output neuron computes a weighted sum of the *R* hidden neuron outputs plus an output bias. Under this formulation, the arithmetic complexity of one forward pass through a single TLP is given by

(4) $$N_{\mathrm{mult}}=R(k+q),$$

(5) $$N_{\mathrm{add}}=R(k+q),$$

(6) $$\;N_{\mathrm{act}}=R,$$

where $N_{\mathrm{mult}}$ is the number of real multiplications, $N_{\mathrm{add}}$ is the number of real additions, and $N_{\mathrm{act}}$ is the number of activation function evaluations. These expressions provide a hardware-agnostic measure of neural network complexity and are later used to compare the literature and proposed models. The activation function evaluations are represented by $N_{act}$. In practical hardware implementations, nonlinear activation functions such as the hyperbolic tangent sigmoid are typically implemented using lookup tables (LUTs) or low-order polynomial approximations. Therefore, the computational cost of activation functions does not primarily correspond to arithmetic operations but rather to memory access and optional interpolation logic.

Since all models in this work are based on the same TLP structure, the main differences in neural complexity arise from the number of input features, the number of hidden neurons, and, in the repeated case, the number of cascaded networks.

### 2.3. Literature Reference Model

The real-valued TLP architecture described in the previous subsections can be employed in different ways depending on how the input signal is represented and how the desired output is constructed. In the literature, several approaches have been proposed to adapt complex-valued PA signals to real-valued neural networks while preserving the relevant nonlinear characteristics of the amplifier.

A commonly adopted strategy consists of decomposing the complex-valued input signal into real-valued features that capture both amplitude and phase information. In particular, the model considered as reference in this work employs the magnitude of the signal and the sine and cosine of phase differences between consecutive samples. This representation allows the neural network to model the AM–AM and AM–PM nonlinearities of the PA while removing carrier frequency information from the signal prior to neural network processing.

The removal of carrier frequency components is necessary because the activation functions used in real-valued neural networks cannot properly reproduce high-frequency oscillations associated with the carrier. Therefore, both the network input and the desired output must be expressed in a baseband representation that excludes the carrier phase. After neural processing, the carrier phase is reintroduced during the output recomposition stage.

Although this approach has been successfully applied in previous PA behavioral modeling studies, the adopted signal decomposition strategy requires several nonlinear mathematical operations, such as magnitude extraction, normalization, and trigonometric computations. These operations significantly increase the arithmetic complexity and hardware implementation cost of the model.

For this reason, the literature model is used in this work as a reference baseline for complexity and performance comparison with the simplified multimode PA model proposed in the following section.

In the literature model, the complex-valued signal decomposition is performed by computing the signal magnitude and the sine and cosine of phase differences between samples. These operations are given by

(7) $$|y_{z}\left(n-m\right)|=\sqrt{ℜ{\left\{y_{z}\left(n-m\right)\right\}}^{2}+ℑ{\left\{y_{z}\left(n-m\right)\right\}}^{2}},$$

(8) $$\cos\left(\theta_{n-m}-\theta_{n-m-1}\right)=ℜ\;\left\{\frac{y_{z}\left(n-m\right)y_{z}^{*}\left(n-m-1\right)}{|y_{z}\left(n-m\right)||y_{z}\left(n-m-1\right)|}\right\},$$

(9) $$\sin\left(\theta_{n-m}-\theta_{n-m-1}\right)=ℑ\;\left\{\frac{y_{z}\left(n-m\right)y_{z}^{*}\left(n-m-1\right)}{|y_{z}\left(n-m\right)||y_{z}\left(n-m-1\right)|}\right\}.$$

**Figure 2:**
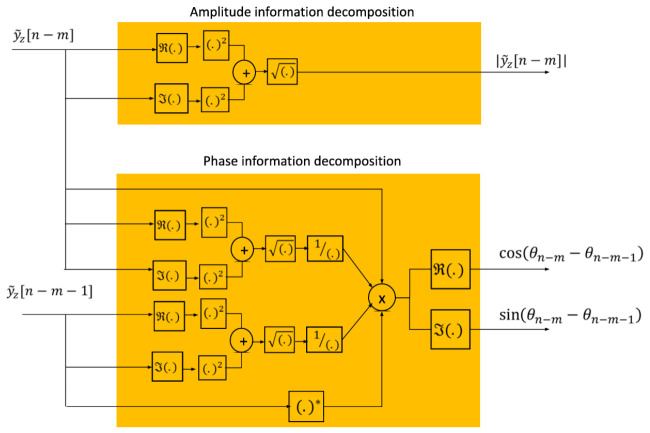
Block diagram of the signal decomposition adopted in the literature real-valued neural network model. The scheme illustrates the mathematical operations required to extract amplitude- and phase-related components while removing carrier-frequency information. Here, ℜ(·) and ℑ(·) denote the real and imaginary parts, ${\left(\cdot \right)}^{2}$ is the squaring operation, $\sqrt{\left(\cdot \right)}$ is the square-root operation, 1/(·) is the reciprocal operation, ${\left(\cdot \right)}^{*}$ denotes complex conjugation, and × represents complex multiplication. The resulting components are the signal magnitude $|{\tilde{y}}_{z}\left[n-m\right]|$ and the phase-difference terms $\cos(\theta_{n-m}-\theta_{n-m-1})$ and $\sin(\theta_{n-m}-\theta_{n-m-1})$.

Here, $\theta_{n}$ denotes the polar angle of the PA input signal at time instant *n*, and $y_{z}\left(n\right)$ represents the complex-valued PA output signal associated with the operating zone *z*. The operator ${\left(\cdot \right)}^{*}$ denotes complex conjugation. This decomposition strategy requires square-root and reciprocal operations, which typically demand dedicated arithmetic units or lookup tables (LUTs) in hardware implementations. As a result, both computational complexity and hardware resource utilization increase. The complete set of mathematical operations required for the input signal decomposition in the literature model is illustrated in Figure 2.

### 2.4. Literature Model Output Reconstruction and Training

In real-valued neural network PA models, the carrier frequency component cannot be directly processed by the network, since the activation functions are not capable of reproducing the high-frequency oscillations associated with the carrier. Therefore, both the input and the desired output signals must be represented in a baseband form where the carrier phase is removed prior to neural processing.

Let $x_{z}\left(n\right)$ denote the complex-valued measured output signal of the PA corresponding to operating mode *z* at discrete time index *n*. In the literature model adopted as reference in this work, the desired output signal used for network training is obtained by removing the instantaneous phase of the input signal. The resulting baseband desired signal is defined as

(10) $${\mathrm{des}}_{\mathrm{lit}}\left(n\right)=x_{z}\left(n\right)e^{-j(\omega t+\theta_{n})},$$

where ω represents the carrier angular frequency and $\theta_{n}$ denotes the instantaneous phase of the input signal at time index *n*. The signal ${\mathrm{des}}_{\mathrm{lit}}\left(n\right)$ therefore corresponds to the PA output expressed in a carrier-free baseband representation.

Since a real-valued neural network is employed, the complex-valued desired signal must be decomposed into two real-valued components before being presented to the network. In this work, these components correspond to the real and imaginary parts of the desired signal, defined as

(11) $${\mathrm{des}}_{\mathrm{lit}}^{I}\left(n\right)=ℜ\{{\mathrm{des}}_{\mathrm{lit}}\left(n\right)\},$$

(12) $${\mathrm{des}}_{\mathrm{lit}}^{Q}\left(n\right)=ℑ\{{\mathrm{des}}_{\mathrm{lit}}\left(n\right)\},$$

where ℜ{·} and ℑ{·} denote the real and imaginary operators, respectively. The signals ${\mathrm{des}}_{\mathrm{lit}}^{I}\left(n\right)$ and ${\mathrm{des}}_{\mathrm{lit}}^{Q}\left(n\right)$ are used as the two real-valued target outputs during neural network training.

After training, the TLP produces two real-valued outputs corresponding to estimates of these components. Let ${\hat{\mathrm{des}}}_{\mathrm{lit}}^{I}\left(n\right)$ and ${\hat{\mathrm{des}}}_{\mathrm{lit}}^{Q}\left(n\right)$ denote the real-valued outputs generated by the neural network. These outputs represent estimates of the baseband desired signal components.

The complex-valued baseband estimate produced by the neural network can therefore be written as

(13) $${\hat{\mathrm{des}}}_{\mathrm{lit}}\left(n\right)={\hat{\mathrm{des}}}_{\mathrm{lit}}^{I}\left(n\right)+j\;{\hat{\mathrm{des}}}_{\mathrm{lit}}^{Q}\left(n\right),$$

where $j=\sqrt{-1}$ denotes the imaginary unit.

Since the carrier phase was removed prior to neural processing, it must be reintroduced to reconstruct the final complex-valued output signal of the model. The reconstructed PA output signal generated by the literature model is therefore obtained as

(14) $${\hat{x}}_{z}\left(n\right)={\hat{\mathrm{des}}}_{\mathrm{lit}}\left(n\right)e^{j(\omega t+\theta_{n})}.$$

**Figure 3:**
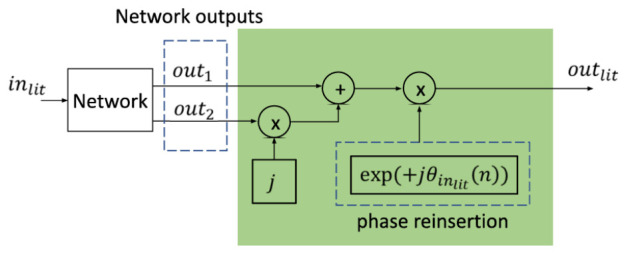
Block diagram of the output signal recomposition stage for the literature model.

Figure 3 illustrates the block diagram of the output recomposition stage used in the literature model.

For the literature model, a single TLP is employed. Therefore, its neural structural complexity is directly obtained from the expressions presented in the TLP Structural Complexity subsection by considering the corresponding number of input features and hidden neurons. In this case, the number of real-valued input features is given by

(15) $$k_{\mathrm{lit}}=3\left(M+1\right),$$

since the input vector contains one magnitude component and two phase-related components for each memory tap.

The parameters of the TLP are optimized during the training stage using a supervised learning procedure. The objective of the training process is to minimize the difference between the desired baseband signal components and the outputs generated by the neural network.

Let ${\hat{\mathrm{des}}}_{\mathrm{lit}}^{I}\left(n\right)$ and ${\hat{\mathrm{des}}}_{\mathrm{lit}}^{Q}\left(n\right)$ denote the real-valued outputs produced by the neural network, corresponding to the estimates of the desired components ${\mathrm{des}}_{\mathrm{lit}}^{I}\left(n\right)$ and ${\mathrm{des}}_{\mathrm{lit}}^{Q}\left(n\right)$, respectively. The instantaneous error signals are defined as

(16) $$e_{I}\left(n\right)={\mathrm{des}}_{\mathrm{lit}}^{I}\left(n\right)-{\hat{\mathrm{des}}}_{\mathrm{lit}}^{I}\left(n\right),$$

(17) $$e_{Q}\left(n\right)={\mathrm{des}}_{\mathrm{lit}}^{Q}\left(n\right)-{\hat{\mathrm{des}}}_{\mathrm{lit}}^{Q}\left(n\right).$$

The training objective is to minimize the mean squared error (MSE) between the desired and estimated outputs. The corresponding cost function is given by

(18) $$J=\frac{1}{N}\sum_{n=1}^{N}\left(e_{I}^{2}\left(n\right)+e_{Q}^{2}\left(n\right)\right),$$

where *N* denotes the number of training samples.

The neural network parameters, including weights and biases, are iteratively updated using the backpropagation algorithm. During this process, the gradients of the cost function with respect to the network parameters are computed and used to adjust the parameters in order to minimize the reconstruction error.

In the literature model, the training procedure assumes that a single neural network can directly learn the nonlinear mapping between the constructed input features and the desired PA output signal. Therefore, the desired signal used during training corresponds directly to the measured PA output after carrier phase removal.

However, in multimode PA operation the measured output signal may contain contributions from multiple nonlinear mechanisms that are not explicitly separated in the training data. In such situations, a single neural network may need to approximate a superposition of nonlinear behaviors, which can increase modeling difficulty.

This observation motivates the development of alternative training strategies capable of progressively isolating different signal components during the learning process. The proposed model introduced in the next section addresses this limitation by employing an iterative cascaded training procedure in which multiple neural networks are trained sequentially while progressively refining the desired signal used in each stage.

## 3. Proposed Simplified Multimode PA Inverse Behavioral Model

From an implementation perspective, the inverse PA model must achieve high accuracy using a limited number of parameters and arithmetic operations. Since DPD operates on high-rate signals, real-time implementation requires low computational latency and reduced memory usage. Consequently, both the signal decomposition stage and the neural network structure must be designed with hardware efficiency in mind.

Motivated by these considerations, this work proposes a simplified real-valued multimode PA inverse behavioral model that reduces the arithmetic complexity of the signal representation while maintaining modeling accuracy. The proposed formulation focuses on simplifying the input feature generation and enabling flexible neural network training strategies suitable for multimode PA behavior.

In the proposed formulation, three parameters define the model structure. The parameter $M_{1}$ denotes the memory depth associated with amplitude-related features, $M_{2}$ denotes the memory depth associated with phase-related features, and $M_{3}$ represents the number of cascaded neural networks used in the repeated training formulation. When $M_{3}=1$, the proposed architecture reduces to a single TLP without cascading.

The simplified signal representation introduced in this work can be combined with different neural network training strategies. In order to isolate the impact of the proposed feature construction and the training procedure, two configurations of the proposed model are considered.

In the first configuration, the proposed simplified signal decomposition is used while maintaining the conventional training procedure adopted in the literature. In this case, a single TLP is trained to approximate the nonlinear mapping between the constructed input features and the desired PA output signal. This configuration allows the impact of the proposed signal representation on modeling complexity and accuracy to be evaluated independently of the training strategy.

In the second configuration, the simplified signal representation is combined with an iterative cascaded training procedure. In this approach, multiple neural networks are connected in cascade, and the desired signal used for training each network is progressively refined by removing the contributions estimated by previously trained networks. This iterative process enables the model to progressively isolate different nonlinear components of the PA behavior.

Therefore, the proposed framework allows two modeling strategies: a simplified single-network model that focuses on reducing the computational complexity of the signal representation, and an iterative cascaded model that further improves modeling capability through sequential neural network training.

The following subsections describe the proposed signal representation and the associated hardware complexity, followed by the iterative cascaded training procedure and the resulting neural network complexity.

### 3.1. Simplified Signal Representation and Feature Construction

The proposed model adopts a simplified signal representation aimed at reducing the computational complexity of the input feature generation stage. Instead of explicitly extracting signal magnitude and phase differences, the proposed formulation directly exploits algebraic relationships between complex samples.

The amplitude-related information is represented using the squared modulus of the complex signal, defined as

(19) $$|y_{z}\left(n-m_{1}\right){|}^{2}=y_{z}\left(n-m_{1}\right)y_{z}^{*}\left(n-m_{1}\right),$$

where $y_{z}\left(n\right)$ denotes the complex-valued PA output signal associated with operating zone *z*, ${\left(\cdot \right)}^{*}$ denotes the complex conjugate, and $m_{1}\in \left[0,M_{1}\right]$ represents the memory index used for amplitude-related features.

The phase-related information is obtained from the complex correlation between consecutive samples, expressed as

(20) $$ℜ\left\{y_{z}\left(n-m_{2}\right)y_{z}^{*}\left(n-m_{2}-1\right)\right\},\;ℑ\left\{y_{z}\left(n-m_{2}\right)y_{z}^{*}\left(n-m_{2}-1\right)\right\},$$

where $m_{2}\in \left[0,M_{2}\right]$ denotes the memory index associated with phase-related features.

This representation avoids explicit magnitude extraction and phase normalization. Consequently, the proposed feature generation relies only on additions and multiplications, eliminating the square-root, reciprocal, and trigonometric operations required by the literature model.

In addition, different memory depths are adopted for amplitude-related and phase-related features. Since these components contribute differently to the dynamic behavior of the PA, separating their memory depths allows the model to reduce unnecessary input expansion while preserving modeling capability. In practice, the smallest memory depth that preserves adequate modeling accuracy should be employed in order to minimize the overall model complexity.

**Figure 4:**
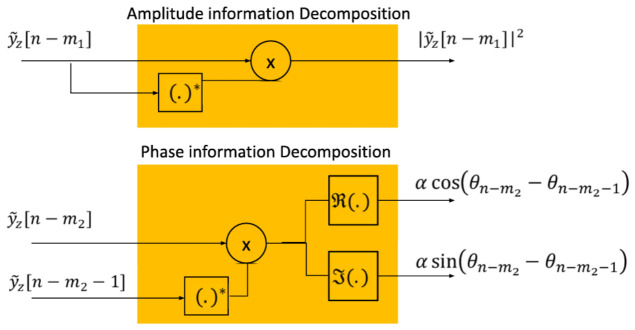
Simplified signal decomposition scheme adopted in the proposed real-valued TLP-based model.

Figure 4 illustrates the mathematical operations required by the proposed signal decomposition method.

### 3.2. Hardware Complexity of Feature Extraction and Signal Reconstruction

To evaluate the implementation cost of the feature extraction and reconstruction stages, the arithmetic and memory requirements of both models were analytically derived.

The analysis is intentionally expressed in a hardware-agnostic manner, considering generic digital processing platforms rather than a specific architecture. Therefore, complexity is characterized in terms of arithmetic operations (multiplications and additions), nonlinear operations (square-root, reciprocal, and trigonometric functions), and memory requirements associated with delay lines and lookup tables.

In typical digital implementations, multiplications are mapped to multiplier or multiply–accumulate units, additions are implemented using combinational arithmetic logic, and memory resources correspond to delay buffers and optional lookup tables used to implement nonlinear functions. Operations such as square-root, reciprocal, and trigonometric functions are commonly implemented using lookup tables, iterative algorithms (e.g., Newton–Raphson), or CORDIC architectures, all of which significantly increase computational and memory complexity.

In addition, practical digital implementations may further optimize arithmetic operations through coefficient-dependent lookup-table representations, distributed arithmetic techniques, or precomputed mappings, particularly in fixed-point or quantized representations. Such strategies can reduce the utilization of dedicated arithmetic units at the expense of additional memory resources and implementation-specific optimization procedures.

Nevertheless, independently of the adopted implementation strategy or target hardware platform, reducing the number of required arithmetic and nonlinear operations inherently contributes to lower hardware resource utilization, reduced memory dependence, and simplified processing pipelines. Consequently, the proposed formulation may also reduce the dependence on LUT/BRAM resources and specialized hardware blocks.

Table 1 summarizes the arithmetic operation count required for the input feature generation stage.

From a hardware perspective, the proposed feature extraction stage may affect both the utilization of logic resources and memory consumption, mainly due to the storage of delayed complex samples, intermediate pipeline registers, arithmetic operators, and data-path organization. Nevertheless, since no practical implementation was carried out in this work, a detailed comparison of memory usage and hardware-resource occupation is outside the scope of this study. These metrics depend on implementation-specific choices, such as numerical precision, pipeline organization, activation-function representation, arithmetic architecture, and the target hardware device [21].

As shown in Table 1, the proposed formulation removes all nonlinear arithmetic operations required by the literature model in both the feature extraction and signal reconstruction stages. Consequently, the front-end processing relies exclusively on additions and multiplications, which are the most efficient operations across digital hardware architectures such as FPGA, ASIC, DSP processors, and dedicated neural network accelerators.

### 3.3. First Proposed Configuration: Simplified Single-Network Model

The first configuration of the proposed framework employs the simplified signal representation introduced in the previous subsection while maintaining the conventional neural network training strategy used in the literature.

In this configuration, a single real-valued TLP is used to approximate the nonlinear mapping between the constructed input features and the desired PA output signal. The input feature vector is composed of amplitude-related terms with memory depth $M_{1}$ and phase-related correlation terms with memory depth $M_{2}$.

Consequently, the total number of real-valued input features is given by

$$k_{\mathrm{prop}}=\left(M_{1}+1\right)+2\left(M_{2}+1\right).$$

The neural network is trained using the same supervised learning procedure described in Section 2, where the network parameters are optimized by minimizing the mean squared error between the network output and the desired signal.

This configuration allows the impact of the proposed simplified signal representation to be evaluated independently of the training strategy.

### 3.4. Second Proposed Configuration: Iterative Cascaded Training Procedure

**Figure 5:**
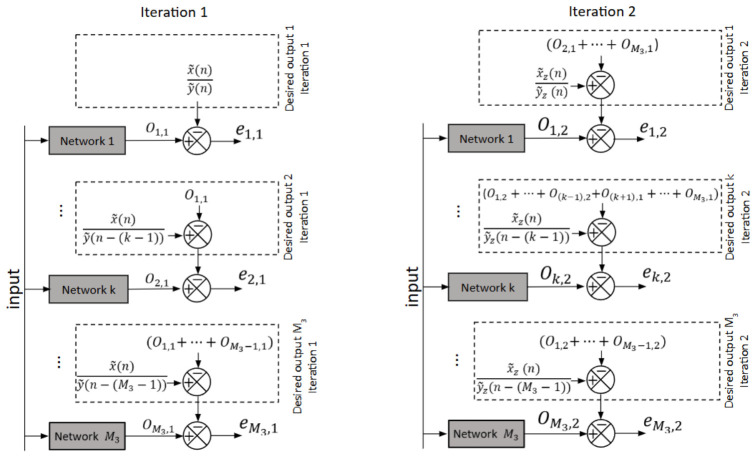
Illustration of the iterative cascaded training procedure used in the proposed model. The desired signal presented to each network is progressively updated by subtracting the contributions of the other cascaded networks.

The second formulation adopts an iterative training strategy in which multiple neural networks are trained sequentially, allowing the model to progressively refine the approximation of the desired signal. The overall training procedure is illustrated in Figure 5. In this approach, each neural network estimates the contribution of a specific delayed input component, while the desired signal presented to each stage is refined by subtracting the contributions estimated by the other networks. The cascaded training strategy adopted in this work is conceptually related to residual learning and residual-refinement techniques previously explored in NN-based DPD and PA behavioral modeling [19,22]. Cascaded NN-DPD modules have also been proposed to improve DPD robustness under varying operating conditions [23]. Therefore, the novelty of the proposed method does not rely on the cascade alone. Rather, the cascade is used as a practical residual decomposition mechanism tailored to the multimode PA inverse modeling problem. Since multimode operation may involve distinct nonlinear and memory-related contributions, the cascaded formulation distributes the overall inverse response across compact TLP stages, instead of relying on a larger single network, with each stage trained to model the residual component not captured by the previous ones.

The proposed cascaded training procedure is described as follows. First, the instantaneous phase of the input signal is removed from the PA output signal according to

(21) $${\tilde{x}}_{z}\left(n\right)=x_{z}\left(n\right)\;y_{z}^{*}\left(n\right),$$

where $x_{z}\left(n\right)$ represents the measured PA output signal and $y_{z}\left(n\right)$ denotes the input signal. This operation produces a phase-aligned target signal that is used during the training procedure.

Let $O_{k,\mathrm{ite}}$ denote the real-valued output produced by the *k*-th neural network at training iteration ite. Each network therefore estimates a coefficient associated with a delayed version of the input signal. After obtaining the network output, the corresponding complex-valued contribution is reconstructed by multiplying the network output by the delayed input signal associated with that stage.

Specifically, the contribution of the *k*-th network is reconstructed as

(22) $$O_{k,\mathrm{ite}}\;y_{z}\left(n-\left(k-1\right)\right).$$

Consequently, the overall complex-valued output of the model at iteration ite is given by

(23) $$O_{\mathrm{model},\mathrm{ite}}=\sum_{k=1}^{M_{3}}O_{k,\mathrm{ite}}\;y_{z}\left(n-\left(k-1\right)\right).$$

During training, the desired signal presented to each neural network is obtained by subtracting the contributions of the other cascaded networks from the phase-removed target signal. Therefore, the desired signal for the *k*-th network at iteration ite can be written as

(24) $$d_{k,\mathrm{ite}}\left(n\right)={\tilde{x}}_{z}\left(n\right)-\sum_{\begin{array}{c} i=1\\ i\neq k \end{array}}^{M_{3}}O_{i,\mathrm{ite}}\;y_{z}\left(n-\left(i-1\right)\right).$$

The error signal used to train the *k*-th network is then computed as

(25) $$e_{k,\mathrm{ite}}\left(n\right)=\left|O_{k,\mathrm{ite}}\;y_{z}\left(n-\left(k-1\right)\right)-d_{k,\mathrm{ite}}\left(n\right)\right|.$$

As illustrated in Figure 5, the training process proceeds sequentially across the cascaded networks and across iterations. At each iteration, the outputs of the previously trained networks are used to update the desired signals of the remaining stages. This procedure progressively isolates the residual nonlinear components that are not captured by the previously trained networks.

The iterative training strategy therefore allows the overall modeling task to be decomposed into multiple simpler sub-problems. Each neural network learns the contribution associated with a specific delayed input component, while the residual modeling error is redistributed among the remaining networks during subsequent iterations.

From a modeling perspective, this procedure leads to a decomposition of the PA behavior in which the output signal is expressed as a weighted combination of delayed input signals with coefficients estimated by the neural networks. The iterative refinement process improves the modeling accuracy without requiring large neural networks, which contributes to reducing the overall computational complexity of the proposed model.

#### Phase Reinsertion

**Figure 6:**
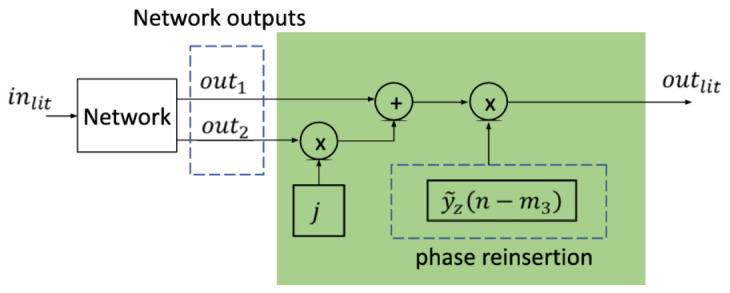
Block diagram of the phase reinsertion stage used to reconstruct the complex-valued output signal from the real-valued neural network outputs.

After the neural network processing stage, the real-valued outputs of the network must be converted back into a complex-valued signal and the phase information removed during the preprocessing stage must be reintroduced. The phase reinsertion procedure adopted in the proposed formulation is illustrated in Figure 6.

The neural network produces two real-valued outputs, denoted as ${\mathrm{out}}_{1}$ and ${\mathrm{out}}_{2}$. These values correspond to the real and imaginary components of an intermediate complex signal obtained after the phase removal step. The first stage of the reconstruction therefore consists of recombining these two components into a complex quantity according to

(26) $$\tilde{o}\left(n\right)={\mathrm{out}}_{1}+j\;{\mathrm{out}}_{2}.$$

Since the instantaneous phase of the input signal was previously removed from the target signal during the preprocessing stage, this phase must be restored in order to obtain the final complex-valued output of the behavioral model. This is accomplished by multiplying the reconstructed complex signal by the delayed input signal used as the phase reference.

Let ${\tilde{y}}_{z}\left(n-m_{3}\right)$ denote the delayed version of the complex input signal associated with the current network stage. The final reconstructed output is therefore obtained as

(27) $$o_{\mathrm{lit}}\left(n\right)=\tilde{o}\left(n\right)\;{\tilde{y}}_{z}\left(n-m_{3}\right).$$

This operation effectively reintroduces the phase of the input signal into the reconstructed output while preserving the amplitude scaling estimated by the neural network. From an implementation perspective, the phase reinsertion stage therefore requires a complex multiplication between the recombined network output and the delayed input signal.

As illustrated in Figure 6, the reconstruction process consists of three main steps: (i) multiplication of the imaginary component by the imaginary unit *j*, (ii) summation with the real component to obtain the complex intermediate signal, and (iii) complex multiplication with the delayed input signal ${\tilde{y}}_{z}\left(n-m_{3}\right)$ to restore the phase information and produce the final complex output.

### 3.5. Neural Network Computational Complexity

The number of real-valued input features of the proposed model is given by

(28) $$k_{\mathrm{prop}}=\left(M_{1}+1\right)+2\left(M_{2}+1\right)=M_{1}+2M_{2}+3.$$

For the proposed model without repetition, a single TLP is employed. In this case, the structural complexity of the network is directly obtained from the expressions presented in the TLP computational complexity subsection by replacing *k* with $k_{\mathrm{prop}}$.

For the proposed repeated model, the architecture is composed of $M_{3}$ TLPs connected in cascade. Consequently, the overall structural complexity scales linearly with the number of cascaded networks. The resulting complexity metrics are expressed as

(29) $$N_{\mathrm{mult}}^{\left(\mathrm{cas}\right)}=M_{3}R\left(k+q\right),$$

(30) $$N_{\mathrm{add}}^{\left(\mathrm{cas}\right)}=M_{3}R\left(k+q\right),$$

(31) $$\;N_{\mathrm{act}}^{\left(\mathrm{cas}\right)}=M_{3}R,$$

where $N_{\mathrm{mult}}^{\left(\mathrm{cas}\right)}$, $N_{\mathrm{add}}^{\left(\mathrm{cas}\right)}$, and $N_{\mathrm{act}}^{\left(\mathrm{cas}\right)}$ correspond to the number of real multiplications, additions, and activation function evaluations required for a single forward pass.

It is important to note that proposed model with iterations ends up increasing the training complexity, but the structural complexity of each individual TLP remains low.

Finally, when $M_{3}=1$, the architecture reduces to a single TLP, since no cascading between networks is required.

Based on the operation counts derived in the previous subsections, the total computational complexity of each model can be expressed as follows.

The total complexity of the literature model is obtained as

(32) $$N_{\mathrm{mult}}^{\left(L\right)}=(9M+11)+4+R(3(M+1)+q)$$

(33) $$\;N_{\mathrm{add}}^{\left(L\right)}=(3M+4)+2+R(3(M+1)+q)$$

(34) $$\;N_{\mathrm{LUT}}^{\left(L\right)}=(4M+5)+2+R$$

For the proposed model without repetition,

(35) $$N_{\mathrm{mult}}^{\left(P_{nr}\right)}=\left(2M_{1}+4M_{2}+6\right)+4+R\left(M_{1}+2M_{2}+3+q\right)$$

(36) $$\;N_{\mathrm{add}}^{\left(P_{nr}\right)}=\left(M_{1}+2M_{2}+3\right)+2+R\left(M_{1}+2M_{2}+3+q\right)$$

(37) $$\;N_{\mathrm{LUT}}^{\left(P_{nr}\right)}=R$$

For the iterative formulation,

(38) $$N_{\mathrm{mult}}^{\left(P_{wr}\right)}=\left(2M_{1}+4M_{2}+6\right)+4M_{3}+M_{3}R\left(M_{1}+2M_{2}+3+q\right)$$

(39) $$\;N_{\mathrm{add}}^{\left(P_{wr}\right)}=\left(M_{1}+2M_{2}+3\right)+2M_{3}+M_{3}R\left(M_{1}+2M_{2}+3+q\right)$$

(40) $$\;N_{\mathrm{LUT}}^{\left(P_{wr}\right)}=M_{3}R$$

These expressions provide the basis for the numerical complexity comparison of the considered models presented in the following section.

## 4. Results

This section presents the linearization performance of the evaluated DPD models, including (i) the literature real-valued TLP approach, (ii) the proposed simplified model without iterative training, (iii) the proposed simplified model with iterative training, and (iv) a baseline scenario without DPD. All results reported in this section refer to the same simulation setup described in the report: 5000 samples, with 3000 used for parameter extraction and 2000 for validation, hyperbolic tangent activation function (tansig), and a single network configured to model all multimode operating regions.

The reported arithmetic and hardware-oriented complexity metrics are directly related to the dimensionality of the considered neural network models. In particular, the number of required operations and nonlinear function blocks scales with the amount of memory, extracted features, and network inputs associated with each formulation.

The results summarized in Table 2 highlight the effectiveness of the proposed real-valued TLP-based DPD models in achieving a favorable trade-off between modeling accuracy and computational complexity. When compared to the literature model, both proposed configurations significantly reduce the number of arithmetic operations and lookup tables required for FPGA implementation, while maintaining comparable NMSE performance. The simplified model without repetition (P_nr) achieves a reduction of approximately 8.16% in multiplications and 65.22% in LUT usage, with no relative NMSE degradation. Further complexity reduction is obtained with the proposed model with iteration training (P_wr), which reduces the number of multiplications by 34.69% and additions by 25.35%, while also improving the NMSE to −42.56 dB. These results confirm that the proposed decomposition and iterative training strategy enable substantial hardware savings without compromising linearization accuracy, making the approach particularly suitable for real-time DPD implementations.

Although the proposed non-iterative model ($P_{nr}$) reduces the number of multiplications and nonlinear operations, Table 2 shows a slight increase in the number of additions. Negative reduction values indicate an increase relative to the literature model. For example, Add. Red. = −14.08% means that the corresponding model requires 14.08% more additions than the literature model. This occurs because the non-iterative formulation relies on a single larger neural network (R=8) to compensate for the absence of iterative refinement. In contrast, the iterative formulation distributes the modeling task among multiple smaller networks, which reduces the number of neurons per network. Since the TLP forward-pass complexity scales with R(k+q), the larger network in the non-iterative case results in more accumulation operations. Nevertheless, this increase has limited hardware impact, as additions are significantly less expensive than multiplications in DSP and FPGA implementations.

**Figure 7:**
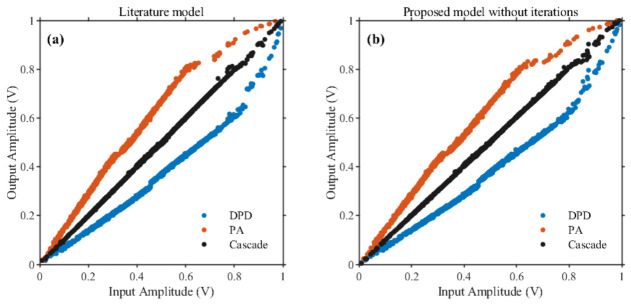
AM–AM characteristics for (**a**) the literature model and (**b**) the proposed model without iterative training.

Figure 7 shows the AM–AM transfer characteristics obtained for the literature model and the proposed model without iterations. In both cases, the cascade response (DPD + PA) becomes closer to the ideal linear behavior compared to the PA response, indicating effective compensation of nonlinear distortion. The proposed approach preserves the linearization capability while employing a simplified real-valued decomposition aimed at reducing hardware complexity.

**Figure 8:**
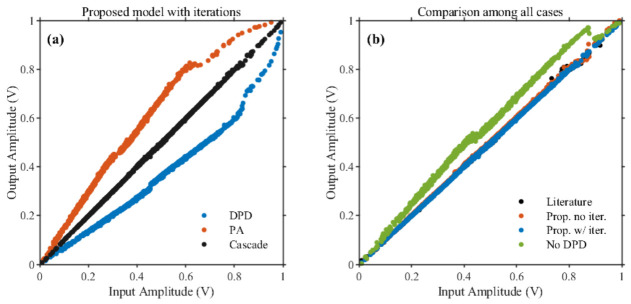
AM–AM characteristics for (**a**) the proposed model with iterative training and (**b**) comparison among all evaluated cases, including the literature model, the proposed models, and the selected no-DPD baseline.

Figure 8 highlights the proposed model with iterative training and a direct comparison among all evaluated cases, including a baseline measurement without DPD. The iterative version further improves the linearization consistency, yielding a cascade response with reduced deviation in the high-amplitude region.

**Figure 9:**
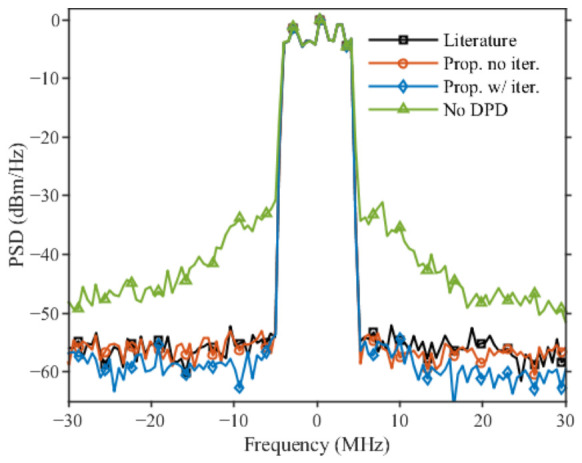
PSD comparison for the literature model, the proposed models with and without iterative training, and the selected no-DPD baseline. The proposed iterative approach provides the best out-of-band suppression.

Figure 9 presents the power spectral density (PSD) comparison for all evaluated cases. The baseline (without DPD) exhibits strong spectral regrowth, whereas all DPD solutions significantly reduce out-of-band emissions. The proposed model with iterative training achieves the lowest adjacent channel power ratio (ACPR), indicating improved suppression of spectral regrowth while maintaining the same average output power.

### Quantitative Metrics

Table 3 summarizes the main performance metrics. The output power is kept approximately constant across all cases, ensuring a fair comparison. The proposed model with iterative training achieves the best ACPR (−55.06 dB) and the best NMSE among the reported extractions (−42.56 dB), outperforming the literature model in both linearity and modeling accuracy.

Overall, the results confirm that the proposed simplifications do not compromise linearization performance. On the contrary, when combined with iterative training, the proposed real-valued approach provides improved ACPR and NMSE while being better aligned with low-complexity implementation constraints.

## 5. Discussion

Two neural network configurations were investigated. The first configuration employs the simplified signal representation together with a single TLP, allowing the impact of the proposed feature construction to be evaluated independently of the training strategy. The second configuration introduces an iterative cascaded training procedure in which multiple neural networks are trained sequentially while progressively refining the desired signal. This approach enables the model to better isolate nonlinear signal components associated with multimode PA behavior.

Experimental results demonstrated that the proposed models achieve modeling accuracy comparable to or better than the reference literature model while significantly reducing implementation complexity. In particular, the cascaded formulation provided the best modeling performance, achieving an NMSE of approximately −42.56 dB while maintaining a reduced arithmetic complexity. Both configurations provide a favorable trade-off between modeling accuracy and computational complexity which represents a major advantage for implementations in FPGA, ASIC, DSP processors, and other digital signal processing platforms.

One of the main advantages of the proposed formulation lies in the simplified signal representation. By replacing magnitude extraction and phase normalization with squared modulus and complex correlation operations, the proposed model eliminates the need for square-root, reciprocal, and trigonometric operations. These operations typically require lookup tables or iterative numerical algorithms when implemented in digital hardware, which increases both computational cost and memory usage. Consequently, the proposed representation demonstrates that equivalent modeling capability can be achieved using simpler algebraic operations focusing on preserving the essential characteristics required for accurate PA modeling.

Another important aspect of the proposed framework is the use of an iterative cascaded training procedure, while the simplified single-network model already provides competitive modeling accuracy, the cascaded configuration further improves the NMSE performance by progressively isolating nonlinear signal components during training. This behavior suggests that the cascaded structure allows the modeling task to be decomposed into simpler sub-problems, each handled by a smaller neural network.

Finally, although the iterative cascaded model improves modeling accuracy, it introduces additional training complexity due to the repeated optimization process. In addition, the present validation remains limited to simulation-based evaluation using the available dataset and analytical operation-count estimates. Although these results are useful to isolate the algorithmic effect of the proposed feature simplification, a complete implementation assessment would require fixed-point design, FPGA or ASIC synthesis, latency measurement, memory-occupation analysis, and real-time validation. Future research may therefore investigate adaptive strategies to automatically determine the optimal number of cascaded networks, explore alternative training procedures to reduce computational cost, and evaluate the proposed formulation using additional datasets covering different modulation formats, signal bandwidths, PA technologies, carrier configurations, and operating points.

## Figures and Tables

**Table 1:** Arithmetic operation count required for input feature generation.

Operation Type	Literature Model (Memory *M*)	Proposed Model ($M_{1},M_{2}$)
Real multiplications	9M+11	$2M_{1}+4M_{2}+6$
Real additions/subtractions	3M+4	$M_{1}+2M_{2}+3$
Square-root operations	M+2	0
Reciprocal/division operations	M+1	0
Trigonometric operations	2(M+1)	0

**Table 2:** Comparison of computational complexity and modeling accuracy for the literature model and the proposed real-valued TLP-based DPD approaches.

Model	*M*	$M_{1}$	$M_{2}$	$M_{3}$	*R*	Mult.	Add.	LUTs	NMSE (dB)	Mult. Red. (%)	Add. Red. (%)	LUT Red. (%)
L	3	0	0	0	4	98	71	23	−40.64	–	–	–
P_nr	0	2	1	0	8	90	81	8	−41.34	8.16	−14.08	65.22
P_wr	0	2	0	3	2	64	53	6	−42.56	34.69	25.35	73.91

Mult.: multiplications; Add.: additions; LUTs: lookup-table-based nonlinear blocks; Red.: reduction relative to the literature model.

**Table 3:** Summary of linearization and modeling metrics for the evaluated approaches.

Method	ACPR (dB)	NMSE (dB)
Literature TLP DPD	−52.43	−40.64
Proposed (no iterations)	−53.11	−41.34
Proposed (iterative training)	−55.06	−42.56
No DPD (baseline)	−33.12	–

## Data Availability

The raw data supporting the conclusions of this article will be made available by the authors on request.

## Abbreviations

The following abbreviations are used in this manuscript:

DPD	Digital Predistortion
PA	Power Amplifier
RF	Radio Frequency
5G	Fifth Generation Wireless
6G	Sixth Generation Wireless
NR	New Radio
3GPP	Third Generation Partnership Project
AM-AM	Amplitude-to-Amplitude Distortion
AM-PM	Amplitude-to-Phase Distortion
PAPR	Peak-to-Average Power Ratio
ANN	Artificial Neural Network
TLP	Three-Layer Perceptron
MLP	Multilayer Perceptron
MP	Memory Polynomial
RVTDNN	Real-Valued Time-Delay Neural Network
NMSE	Normalized Mean Square Error
MSE	Mean Square Error
ACPR	Adjacent Channel Power Ratio
PSD	Power Spectral Density
FPGA	Field Programmable Gate Array
ASIC	Application Specific Integrated Circuit
DSP	Digital Signal Processor
LUT	Lookup Table
I/Q	In-phase and Quadrature
